# Sweat exosomes: A new horizon of liquid biopsy in cancer

**DOI:** 10.1016/j.jlb.2023.100122

**Published:** 2023-10-16

**Authors:** Divya Mirgh, Anand Krishnan, Sukhamoy Gorai

**Affiliations:** Vaccine and Immunotherapy Center, Massachusetts General Hospital, Boston, USA; Department of Chemical Pathology, School of Pathology, Faculty of Health Sciences, University of the Free State, Bloemfontein, 9300, South Africa; Department of Neurological Sciences, Rush University Medical Center, Chicago, IL, USA

Dear Editor,

Extracellular vesicles have added a new era to the cancer liquid biopsy approach. It originates from living cells (majorly classified into microvesicles, apoptotic bodies, and exosomes) and is found in all biofluids (blood, plasma, serum, urine, cerebrospinal fluid (CSF), saliva, sweat, etc.). The main composition of sweat consists of electrolytes, metabolites, proteins, cytokines, and hormones [1]. It can be said that there is a difference in sweat composition in the normal state compared to the disease state, which can be observed as a biomarker of that disease [2]. Exosomes are secreted by a wide range of cells, whether in normal or diseased states. These tiny vesicles transport active biomolecules such as DNA, RNA, and proteins. Furthermore, they play a pivotal role in facilitating intercellular communication within complex biological systems [3]. The simplicity of the sweat matrix is advantageous in the search for biomarkers to study diseases as it is less susceptible to interference from other pathological conditions compared to more intricate samples [4]. Lung cancer can be screened with the help of sweat analysis. Sweat exosome-based lung cancer detection possible in clinical samples (based on exosomes molecular expression) [5]. Two known sweat biomarkers that are used for cancer prognosis are Dermcidin (DCD) and Prolactin Inducible Protein (PIP). DCD overexpression can be seen on cell surfaces of breast carcinomas and lymph nodes while PIP overexpression is observed in exocrine tissues of breast and prostate cancer [6]. Additionally, exosomes have the capability to transform the recipient cell's activity via the transport of biologically active cargo. This molecular signature supports the detection of diseases. Moreover, they actively participate in modulating disease progression, especially in the context of cancer [3]. Exosomes released by Tumor (tumor-derived exosomes-TEXs) [11] cells have garnered attention as highly potential markers for cancer diagnosis because of their distinctive makeup and roles. Detecting and measuring specific exosomes, even in minute clinical sample volumes, can serve as a noninvasive method for both diagnosing and predicting the course of cancer [7]. The biomarkers present in sweat can be assessed by using the combination of gas chromatography and mass spectrometry which further permits us to analyze the volatility of sweat among different populations [1]. Researchers have shown significant interest in exosomes originating from tumors due to their extensive involvement in various stages of tumor formation. These exosomes play a pivotal role in promoting both the growth and spread of tumors by facilitating communication between cells. Additionally, because of the unique surface proteins they possess, exosomes are considered valuable diagnostic biomarkers for diseases [8]. Sweat exosomes and electronano chemistry based sensor development is a mega initiative of the next generation of cancer diagnostic tool development. In exosome research most complicated domain is exosome heterogeneity [9] (it can be solved via a single exosome profiling approach) if we properly address this limitation, exosomes be a promising platform for future precision oncology [10]. Hope this article encourages research minds to develop a smart approach against global cancer burdens.

## Declaration of competing interest

The authors are declaring no conflict of interests.
